# Accurate and Automatic Extraction of Cell Self-Rotation Speed in an ODEP Field Using an Area Change Algorithm

**DOI:** 10.3390/mi13060818

**Published:** 2022-05-24

**Authors:** Haiyang Wu, Dan Dang, Xieliu Yang, Junhai Wang, Ruolong Qi, Wenguang Yang, Wenfeng Liang

**Affiliations:** 1 School of Mechanical Engineering, Shenyang Jianzhu University, Shenyang 110168, China; haiywu@foxmail.com (H.W.); yang.xieliu@sjzu.edu.cn (X.Y.); jhwang@sjzu.edu.cn (J.W.); 2 School of Science, Shenyang Jianzhu University, Shenyang 110168, China; 3 School of Electromechanical and Automotive Engineering, Yantai University, Yantai 264005, China; yangwenguang@ytu.edu.cn

**Keywords:** ODEP, self-rotation, area change

## Abstract

Cells are complex biological units that can sense physicochemical stimuli from their surroundings and respond positively to them through characterization of the cell behavior. Thus, understanding the motions of cells is important for investigating their intrinsic properties and reflecting their various states. Computer-vision-based methods for elucidating cell behavior offer a novel approach to accurately extract cell motions. Here, we propose an algorithm based on area change to automatically extract the self-rotation of cells in an optically induced dielectrophoresis field. To obtain a clear and complete outline of the cell structure, dark corner removal and contrast stretching techniques are used in the pre-processing stage. The self-rotation speed is calculated by determining the frequency of the cell area changes in all of the captured images. The algorithm is suitable for calculating in-plane and out-of-plane rotations, while addressing the problem of identical images at different rotation angles when dealing with rotations of spherical and flat cells. In addition, the algorithm can be used to determine the motion trajectory of cells. The experimental results show that the algorithm can efficiently and accurately calculate cell rotation speeds of up to ~155 rpm. Potential applications of the proposed algorithm include cell morphology extraction, cell classification, and characterization of the cell mechanical properties. The algorithm can be very helpful for those who are interested in using computer vision and artificial-intelligence-based ideology in single-cell studies, drug treatment, and other bio-related fields.

## 1. Introduction

With the intensive research into micro and nano mechanics and the rapid advancement of microscopy, cellular level research has become a hot topic in many fields. As the most basic unit of life in higher organisms, cells can not only grow, develop, proliferate, age, and die independently in an adapted environment, but they can also organically form tissues and organs with different morphologies and functions in order to maintain the functions necessary for life, thereby forming a complete living organism [1]. Cells have specific tissue and organ functions, and are therefore regarded as single units to examine and analyze the multidimensional characteristics of cells during proliferation, differentiation, movement, and other physiological activities. This is significant for revealing the complex and diverse changes in living bodies, as well as their distribution patterns in time and space [2,3]. The multidimensional information of cells includes cellular morphological information, electrophysiological information, and mechanical properties, among others [4,5,6,7,8,9,10]. The mechanical properties of cells play a pivotal role in characterizing different cellular states and functions. Cells live in a complex mechanical and chemical environment where changes in the physical mechanics of the cell activate cellular signaling channels and induce reorganization of the cytoskeleton [11]. At different levels of the cellular hierarchy, changes in the cellular organization can be characterized by changes in the specific mechanical properties. Clearly, cells of different structures vary greatly in several aspects, including biological activities, such as cell differentiation, growth, and adhesion, and the pathogenic mechanisms of life, such as oxidative stress, viral attack, and parasites of life [12,13]. Thus, quantifying the mechanical properties of living cells provides information about the actual conditions of the cells. To understand how cells respond to their surroundings, it is necessary to characterize their mechanical properties. Researchers at the University of Texas discovered, for the first time, that changes in the mechanical properties of cells may be responsible for tumorigenesis [14]. Changes in the mechanical properties of cells will eventually lead to uncontrolled cell division, and such uncontrolled cells have a low mortality rate and give rise to the growth of malignant tumors. Plenty of research on cancer has shifted focus to the biochemical perspective in order to address the various interdependent factors associated with biochemical carcinogenesis. Meanwhile, the mechanical properties of cells affect their ability to be deformed, to be moved, and to perceive external stimuli in their microenvironment. Research has shown that changes in the mechanical properties of cells define the direction for the investigation of many cellular diseases [15]. New techniques for the precise capture and manipulation of cells at a microscale have also made it possible to characterize the mechanical properties of cells.

Several techniques are currently available for cell manipulation and are being used in different fields, such as optical, ultrasound, magnetic, and electric fields [16,17,18,19,20,21,22]. Optical tweezers, which use lasers to generate forces to trap cells, require high-intensity light. This method has several limitations. Specifically, the cells may easily become damaged; the applied range is limited by the area in which the optical tweezer system can operate, which impedes the manipulation of cells in large quantities. Meanwhile, acoustic forces generated using ultrasound can be used to trap or lift cells. Nowadays, it is commonplace to manipulate cells using electrical forces, such as dielectrophoresis (DEP) [23,24,25]. A new technique for manipulating tiny particles based on DEP forces, also known as optically induced dielectrophoresis (ODEP), was first introduced by Ming et al. [26]. ODEP can realize label-free and non-invasive manipulation of cells [27,28] and microparticles [29,30] through optically projected patterns serving as virtual electrodes. Hence, fabricated metal electrodes for generating a non-uniform electric field in DEP are not required [31]. In addition, ODEP can manipulate individual cells or particles, which is not easy for DEP [32]. ODEP combines the advantages of optical tweezers and DEP for the manipulation, sorting, organization, and pattern formation of micro and nano particles, offering a powerful technique for optical manipulation. Subsequently, by combining microfluidic, electrophoretic, and microscopic imaging techniques in a rotating chamber, L. Huang et al. generated a rotating electric field by applying an appropriate alternating current (AC) signal to the electrodes to drive controlled three-dimensional (3D) rotation of a single cell, enabling 3D cell imaging [33]. The area-specific membrane capacitance and cytoplasmic conductivity of different cell types were determined through tests. The 3D cell imaging of cancer cells and normal leukocytes demonstrated that there were subtle differences in the geometric parameters of the two cell types. Some cells with specific intrinsic dielectric properties can also self-rotate in a linearly polarized (i.e., non-rotating) AC field. For example, the self-rotation of Melan-A pigment cells can be induced through a specific optical electrode pattern that generates ODEP forces and a bandwidth of AC bias frequencies [34]. The self-rotation phenomenon is used to obtain the behavioral information of pigment cells so as to elucidate their physical properties, which in turn allows for the separation of pigment cells from non-pigment ones. Our group proposed a method that can extract the mechanical properties of cells by manipulating Raji cell translation and self-rotation through DEP/ODEP-based microfluidics [32,35]. An advantage of this method is that it allows individual cells to rotate in a known and controlled direction in a rotation-free electric field, without any requirement on the threshold of the electric field. This method opens up further possibilities for the label-free identification of cancer and related clinical applications. This method achieves rotational control of the cells, but relies on rotation speed data to obtain the final measurements of the physical properties. Usually, the rotation speed of cells is measured visually by manually timing their rotation under a video microscope using a stopwatch [33,35]. However, manual measurement can be tedious and prone to large errors. Therefore, an automated computer-vision-based method is required to identify the morphology of the cell accurately and quickly, and to measure its rotation speed. A number of cell rotation speed extraction methods have been proposed by various research groups. For example, a computer-based real-time machine vision algorithm was first proposed by G. De Gasperis et al. for measuring cell rotation and analyzing rotational spectra [36]. However, this method has the limitation that it can only measure in-plane rotation when only a single cell is present in the image. A multi-cell visual tracking algorithm capable of achieving an automatic process for determining cell information has been reported [37]. The algorithm uses the Sobel operator to extract horizontal and vertical gradients in order to identify cells in a computationally cumbersome manner. It is difficult for this proposed algorithm to obtain out-of-plane rotating cell speeds [37]. Our group proposed an automated algorithm based on image matching that can accurately determine the rotation speed of cancer cells in an ODEP-based microfluidic chip [27,38,39,40,41,42]. An optical-flow-based method was proposed in order to obtain 2D kinematic field data from the image sequences. Then, the data were back-propagated onto a 3D sphere model to calculate the rotation axis and rotation speed of the cell [43]. Both methods are effective at measuring the self-rotation speed of cells in the ODEP field, but the algorithms are too complex and computationally intensive. To improve the automation and efficiency of the cell self-rotation speed extraction, we recently developed a new area change algorithm to overcome the limitations of the existing methods. Specifically, this algorithm provides a simplified process for cell self-rotation speed extraction compared with previous algorithms. In addition, this algorithm employs three key techniques to achieve the following goals: (1) It reduces the influence of experimental conditions on the measurement results by removing vignettes and improving the contrast of cells, and it can extract a complete cell morphological profile. This demonstrates the good adaptability of the algorithm. (2) After selecting cells of interest in the first frame, the algorithm calculates the area of the cells, regardless of whether they are rotating in-plane or out-of-plane and without interference from other cells within the image. This eliminates the need to manually observe the video, and thereby reduces the reliance on the manual intervention for results and ensures higher stability. (3) The algorithm denoises the area change curve using a convolution method, allowing for more accurate extraction of the cell rotation period and more accurate measurements of the cell rotation speed.

In this paper, we proved that the self-rotation of cells can be characterized automatically, efficiently, and accurately through captured videos of multiple cells experiencing different motions using a new area change algorithm. We presented the key steps involved in the proposed algorithm in detail, including outline extraction and speed calculation based on the microscopic images of cells. We first calculated the self-rotation speed of Raji cells under a given AC bias parameter to demonstrate the specific calculation process. Then, this process was repeated to calculate the self-rotation speed of Raji cells under several other AC bias parameters.

## 2. Theory

In general, ODEP makes use of the light-sensitive properties of the low-conductivity optical semiconductor material to generate an electric field perpendicular to the chip between the upper and bottom electrodes of the chip. The structure of the electrodes is determined by the incident optical pattern. In addition, the optical semiconductor material has an extremely high resistance in the dark region, and most of the applied voltage falls on the hydrogenated amorphous silicon (a-Si:H) layer. The optical semiconductor material generates a large number of optically induced carriers in the lighted region, thus sharply increasing the local conductivity. When a voltage is applied, different partial pressures are generated in the bright and dark regions, so that an inhomogeneous electric field is created between the upper and lower pole plates of the chip. If suspended particles are in close proximity to this inhomogeneous electric field, the interaction between the two particles and the electrically polarized dipole moment of the liquid solution generates a force, which is called the DEP function and is defined as ODEP. Generally, when the polarization capacity of the particles is greater than that of the solution, the particles are induced to move to a higher electric field gradient by generating a positive DEP force. On the contrary, a negative dielectrophoretic force is induced to move to a lower electric field gradient. This means that DEP forces can be either positive or negative. In the case of an inhomogeneous electric field, the DEP force on a spherical particle can be expressed as [44].

(1) 〈F→DEP〉=2πR3εmReKω∇E→rms

where *E_rms_* is the square root mean of the electric field strength; *R* is the cell radius; *ε_m_* denotes the dielectric constant of the liquid medium; *ω* is the angular frequency with the expression *ω* = 2π*f*, where *f* is the frequency of the voltage applied across the liquid medium; and Re [*K (ω)*] is the real part of the Clausius−Mossotti (CM) factor that determines the direction of the DEP force. For cells, the expression for Re [*K (ω)*] is usually written as

(2) ReKω=Re−ω2τ1τ2−τcτ2′+jωτ2′−τ1−τ2−1ω2τcτ2′+τ1τ2−jωτ2′+2τ1+τ2−2

where τ_c_ = ε_c_/σ_c_, τ_1_ = ε_m_/σ_m_, τ_2_ = C_mem_/σ_c_, and τ_2_′ = C_mem_/σ_m_; the subscripts c, mem, and m denote the cytoplasm, cell membrane, and liquid medium, respectively; C_mem_ denotes the capacitance value of the cell membrane; and *ε* and *σ* denote the dielectric constant and conductivity, respectively. When the electric field frequency approaches zero, the size of the real part of the CM factor is mainly affected by the conductivity of the solution and the particles. On the contrary, when the electric field frequency is very high, the real part of the CM factor is only affected by the dielectric constant. If the positive and negative values of the CM factor are known at different frequencies, the particles can be manipulated by modulating specific frequencies to produce positive and negative DEP forces.

In terms of cell manipulation, previous studies have not only reported the translational motion of cells in ODEP fields, but also demonstrated that certain types of cells self-rotate in linearly polarized AC fields along an axis perpendicular to the electric field lines [27,42]. Turcu et al. proposed that a theoretical analysis can explain the fact that certain cells with specific dielectric properties can self-rotate in a non-rotating electric field [45]. Based on previous studies, our group attempted to describe the rotational behavior of colored cells with different contents of intrinsic melanin in a linearly polarized AC electric field [35]. We also experimentally confirmed the rotation phenomenon by implanting foreign particles into the cells. Prior to inoculation, these cells did not initially rotate in the presence of an externally applied non-rotating AC electric field. In general, the rotation equation is defined as

(3) $$T=\frac{9}{4}V\varepsilon_{1}E_{0}^{2}\frac{\varepsilon_{r}-\sigma_{r}}{\left(\varepsilon_{r}+2\right)\left(\sigma_{r}+2\right)}\left(\frac{X+X_{0}}{{\left(X+X_{0}\right)}^{2}+1}+\frac{X-X_{0}}{{\left(X-X_{0}\right)}^{2}+1}\right)$$

where *V* is the volume of cells. The other symbols are as follows:

(4) $$\left\{\begin{array}{l} \varepsilon_{r}=\varepsilon_{p}/\varepsilon_{m}\\ \sigma_{r}=\sigma_{p}/\sigma_{m}\\ \omega_{0}\tau =X_{0}\\ \omega \tau =X\\ \tau =\left(\varepsilon_{p}+2\varepsilon_{m}\right)/\left(\sigma_{p}+2\sigma_{m}\right) \end{array}\right.$$

where *ω_0_* is the angular frequency of the cellular motion. From the above equation, we can clearly conclude that the electrophysiological information and mechanical properties of the cell can be extracted from its behavioral information, as the self-rotation varies with frequency.

## 3. Materials and Methods

### 3.1. Experimental Setup and Working Principles

**Figure 1:**
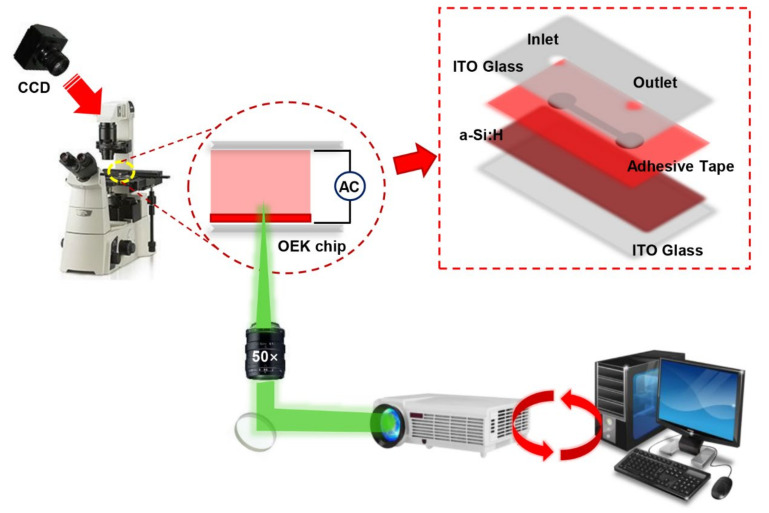
Schematic illustration of the ODEP-based microfluidic platform used as the experimental setup. The inset is an exploded view of the ODEP chip.

An ODEP-based microfluidic platform was designed as the experimental setup in this study. Its structure is shown schematically in Figure 1. The system has been described in detail in our previous work [42]. We used a CCD camera to record the movement of cells in the ODEP chip as an image injected through a charge-coupled projector. The ODEP chip consists of four layers from top to bottom. The top layer is a transparent film of indium tin oxide (ITO) glass; the middle part is a custom-designed microchannel with a hydrogenated amorphous silicon (a-Si:H) layer, which is connected to the top layer by a fabricated tape; and the bottom part is an ITO glass substrate with a hydrogenated amorphous silicon (a-Si:H) film deposited on top.

When the designed optical pattern was projected onto the a-Si:H surface, the electron−hole pairs were excited and enhanced by the migration of electrons from the valence band to the conduction band of the a-Si:H layer, resulting in a large number of optically induced carriers and a significant increase in local conductivity. When a voltage was applied, different partial voltages were generated in the light and dark regions, which in turn created an inhomogeneous electric field between the upper and lower pole plates of the chip. The electric field at the end of the liquid chamber then increased sharply above the locally illuminated a-Si:H region, as most of the applied voltage was shifted sharply into the liquid chamber. As a result, an inhomogeneous non-rotating electric field was generated in the liquid chamber.

### 3.2. Cell Preparation

The cells used in the experiments were Raji cells bought from the Cell Bank of the Chinese Academy of Sciences (Shanghai, China). They were suspended in the cell culture medium supplied by the Roswell Park Memorial Institute (RPMI1640, Thermo Fisher Science, Bridgewater, NJ, USA), which contained 10% (*v*/*v*) fetal bovine serum, 1% penicillin (*v*/*v*) (100 U/ml), and 1% streptomycin (*v*/*v*) (100 µg/ml), and were incubated at 37 °C in a humidified atmosphere of 5% CO_2_. The diameter of the Raji cells was about 12 µm.

Before the experiment, 1 mL of the Raji cell suspension was centrifuged at 1000 rpm for 5 min at 4 °C and then the supernatant was removed. The collected Raji cells were resuspended in 1 mL of RPMI-1640 culture medium and centrifuged again with the same parameters to remove the remaining culture medium. The remaining Raji cells were then resuspended in 1 mL of isotonic solution for further experiments. The isotonic solution was prepared from 8.5% (*ω*/*v*) sucrose, 0.3% (*ω*/*v*) glucose, and 0.5% (*ω*/*v*) bovine serum albumin (BSA) in deionized water. The purpose of using BSA was to reduce the affinity between the cells and the a-Si:H substrate. The conductivity of the isotonic solution was measured using a conductivity meter (Cond3110, VWR International, RADNOR, PA, USA) at 1.5 × 10^−2^ S/m. The cell concentration of the cell suspension was kept constant at 1 × 10^5^ cells/mL.

### 3.3. Self-Rotation Speed Extraction

Generally speaking, the projected area between each frame captured by the CCD camera varied as the cells rotated in an ODEP field. Therefore, a curve of cell area changes could be obtained by measuring the area of the rotating cells in all of the frames. As the images captured by the camera contained multiple cycles of cell rotations, the cell area change curve varied periodically. Moreover, the time required to perform a complete rotation could be obtained by selecting the trough points of adjacent cycles in the cell area change curve, i.e., the frames with the smallest area points in the adjacent rotation cycles. Therefore, the cell area change curve is a reliable method for obtaining the rotation speed of the cell.

**Figure 2:**
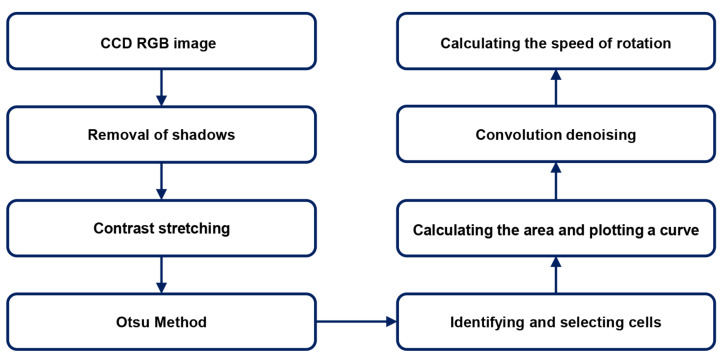
Flow chart of the algorithm for extracting cell speed based on area change.

Considering the characteristics of the cells, the imaging conditions, the low contrast between cells and the image background, the unclear cell edges, and the presence of impurities such as regionally brighter light sources and broken incomplete cells in the image background, a fluorescent microscope was used to capture the experimental images under poor lighting conditions. Through image processing, a complete and smooth cell outline was efficiently segmented without destroying the integrity of the characteristic points inside the cells. In addition, the self-rotation speed of the self-rotating cells in the cell physical field was extracted efficiently, automatically, and accurately by extracting the morphological characteristics of the cell from the 2D cell microscopic images. This algorithm contains three important steps for achieving more accurate and faster speed extraction. First, in order to extract the cell outline completely, the original cell image was vignetted to reduce errors in cell image segmentation, thus improving the accuracy of area calculation. Second, the rotating cells were selected, and the areas of those cells in each frame of the image were extracted and used to plot a cell area change curve. Third, the cell area change curve was denoised to obtain a periodically changing curve. The specific steps are described in Figure 2.

#### 3.3.1. Removing shadows

**Figure 3:**
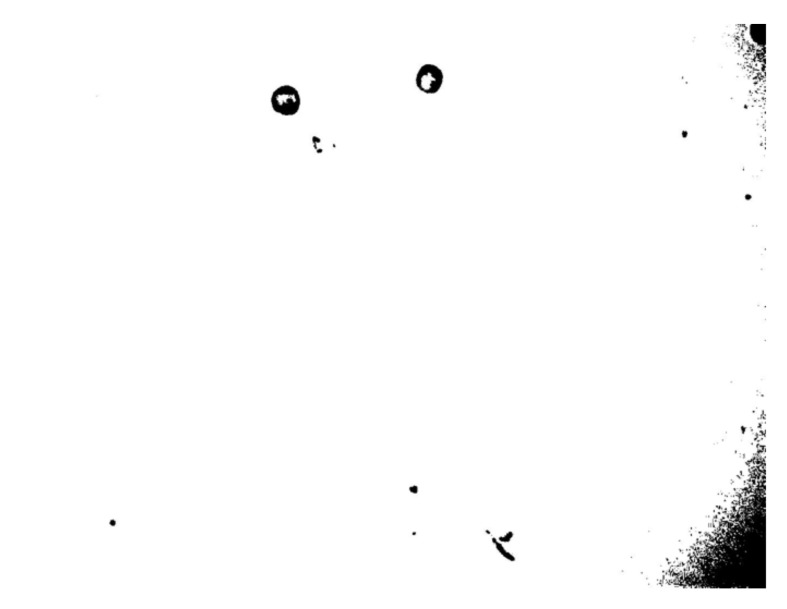
Binarization of cell images without removing the dark corners.

As a result of the presence of a light source in the center of the image, the corners of the image were less bright or saturated than the middle part of the image. The dark shadows in the corners of the image needed to be removed, so as to minimize the noise in the image after binarization of the final segmentation, as shown in Figure 3.

The first method we used was shadow correction based on entropy minimization [46]. Minimizing the entropy of the whole image has also been proven to be an effective method for shadow correction. However, the optimization process could cause the ordinary entropy to fall into a local optimum. Therefore, according to the concept of logarithmic entropy, if the histogram of an original normal image has a single-peaked distribution, then for an image with dark corners, there must be another distribution of low luminosity in the histogram. The purpose of correcting dark corners is to make the distribution of low luminosity closer to the original normal luminosity. Ordinary entropy calculations will not decrease until there is a partial overlap between the two histograms. Before this, the entropy is always increasing, while the logarithmic entropy at least remains constant until there is no overlap. This, in turn, offers a better way to obtain a global optimum. The luminance is first mapped logarithmically and the mapping equation is as follows

(5) $$i\left(L\right)=\left(N-1\right)\log\left(1+L\right)/\log256$$

where L is the input image of cells, i is the grey-scale value, and N is the pixel value.

The first method has a slightly slower calculation speed, and typically takes 15,000 ms to process a 1392 × 1040 color map. Therefore, we used gamma correction as the second method [47]. As the gamma curve of the image was edited for non-linear tone editing of the image, the dark and bright parts of the image signal were detected and the ratio of the two was increased to improve the image contrast. Then, gamma correction enhanced the storage accuracy of the dark luminance, i.e., it removed the dark parts by increasing the luminance of the dark corners.

(6) $$f\left(I_{output}\right)={I_{input}}^{\gamma }$$

The value of *γ* determines how the grey scale is mapped between the input image and the output image, i.e., whether the contrast in the lower or higher grey-scale areas is enhanced. If *γ* > 1, this indicates enhanced contrast in the higher grey-scale areas of the image; if *γ* < 1, this indicates enhanced contrast in the lower grey-scale areas of the image. This results in a brightness-enhanced image.

#### 3.3.2. Enhancing contrast

**Figure 4:**
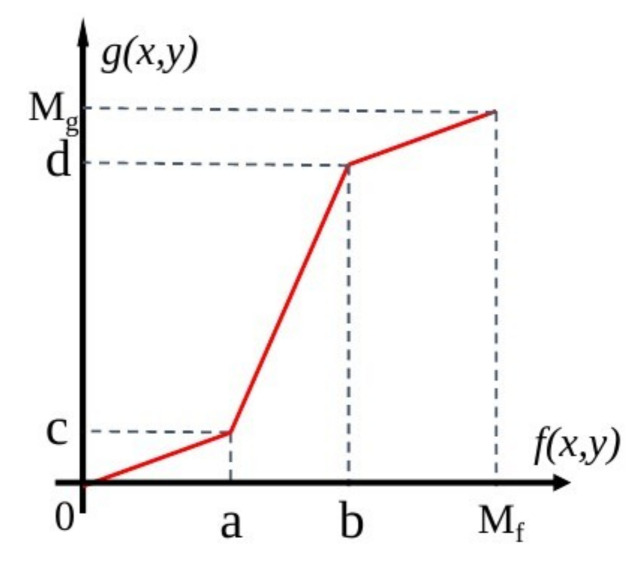
Grey-scale stretching curve.

As a result of the low contrast between the cells and the background and the presence of brighter noise within the image, a segmented linear method was used to highlight the targets or grey-scale intervals of interest and suppress grey-scale areas not of interest. The grey-scale interval [a, b] was extended, and the grey-scale intervals [0, a] and [b, M_f_] were compressed (as shown in Figure 4). By adjusting the position of the fold inflection points and controlling the slope of the segmented straight lines, the grey-scale intervals of interest could be extended. Ultimately, the parameters were adjusted to enhance the contrast between the cells and the background and to reduce the contrast between the light source and the background, thereby allowing for clearer observation of the cell outline.

As a result of the generally low concentration of grey values in the pixels, the whole image can be very dark and not quite as contrasted. With grey-scale transformation, as shown in Equation (7), we stretched the grey values to a specified interval to enhance the contrast significantly.

(7) $$I\left(x,y\right)=\frac{I\left(x,y\right)-I_{\min}}{I_{\max}-I_{\min}}\left(MAX-MIN\right)+MIN$$

where *I_max_* and *I_min_* are the minimum and maximum grey values of the original image, respectively. *MAX* and *MIN* are the minimum and maximum grey values of the grey space, respectively.

#### 3.3.3. Binarization

After linear stretching of the image contrast, the morphological characteristics of the cells could be distinguished more clearly. At this point, the Otsu method was used for binarization [48]. The image was segmented into two parts—the background and the foreground—according to the grey-scale characteristics of the image. As variance is a measure of the uniformity of the grey-scale distribution, the greater the interclass variance between the background and foreground, the greater the difference between the two parts of the image. When part of the foreground was mis-segmented into the background or part of the background was mis-segmented into the foreground, the interclass variance between the two parts became smaller. Therefore, the segmentation that resulted in the largest interclass variance had the lowest probability of misclassification [49]. The Otsu method is less affected by image brightness and contrast, which can further reduce the effect of low contrast in dark parts on the segmentation results.

#### 3.3.4. Post-Processing

As observed from the binarized image, the cell outline was not smooth and complete, and there were still some impurities around the cells. Therefore, the image needed to be post-processed. Using a morphological approach, objects with small areas in the binary image were removed to reduce the excess noise. In addition, an expansion operation was performed to smooth out the cell outline. Finally, a complete and smooth cell outline was obtained.

#### 3.3.5. Extraction of Cell Behavior

After the previous morphological manipulation, narrow junctions in the binary image were disconnected, small protrusions were eliminated, and cell contours were smoothly extracted. All adjacent white pixels in the binary image were labelled based on the traversal of the image, and pixels with the same marker formed a connected domain. White pixels in different connected domains were labelled differently so that each connected domain in the image could be extracted. Finally, all cells in the resulting binary image were identified and labelled using an external rectangle to frame the connected domains and a shape center to determine the location of the connected domains. A cell of interest was selected and extracted separately. Its area was calculated using a morphological method. When the cell was rotated in-plane, the smallest outer rectangular box of the cell was determined and the cell area within a quarter of the box was extracted. The cell rotation period was determined by the change in cell area within this section.

This experimental image had 159 frames. The above operation was performed on each frame to obtain an area change curve of this cell projected onto the 2D plane as it self-rotated in the physical field. From the area change curve, it could be tentatively determined that the area of the cell varied periodically, i.e., the speed of its self-rotation could be determined by the area change. To obtain a clearer period, the area change curve was smoothed and denoised to filter out the excess peaks using a convolution method.

Finally, the self-rotation speed was extracted through the processed area change curve. When the cells were rotated out-of-plane along a fixed axis, the area of the cell image varied at different moments during one rotation cycle. Then, the trough of a cell area change cycle was labeled where the cell area reached a minimum value. When the cell area shifted to the trough point of the next cycle, the cell would rotate for a complete week. The final rotation speed was calculated using Equation (8).

(8) $$n=\frac{60f_{\mathrm{fps}}}{X_{i}-X_{i-1}}$$

where ƒ_fps_ denotes the frame rate of the video, ƒ_fps_ =15 fps; *X_i_* and *X_i-1_* denote the frame numbers of the wave valley points in two adjacent cycles of area change; and n is the average self-rotation speed (in rpm).

In addition, for extracting the displacement information of the cells, the frame difference method was used to detect the moving targets in each frame of the image. Tracking of the moving targets was achieved using a time-series-based prediction model and the displacement trajectory of the cell was plotted.

## 4. Results and Discussion

### 4.1. Self-Rotation Speed of Raji Cells under a Given AC Bias Parameter

**Figure 5:**
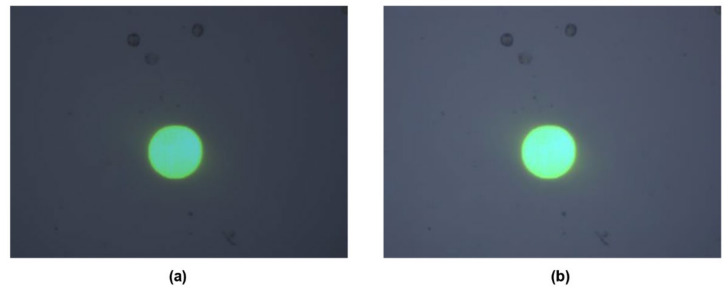
(**a**) Original cell image taken by CCD and (**b**) cell image with dark corners removed.

**Figure 6:**
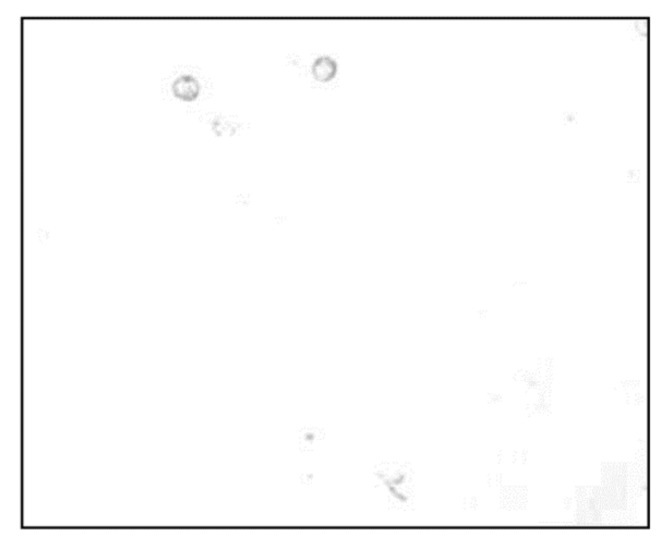
Grey-scale image after contrast stretching.

**Figure 7:**
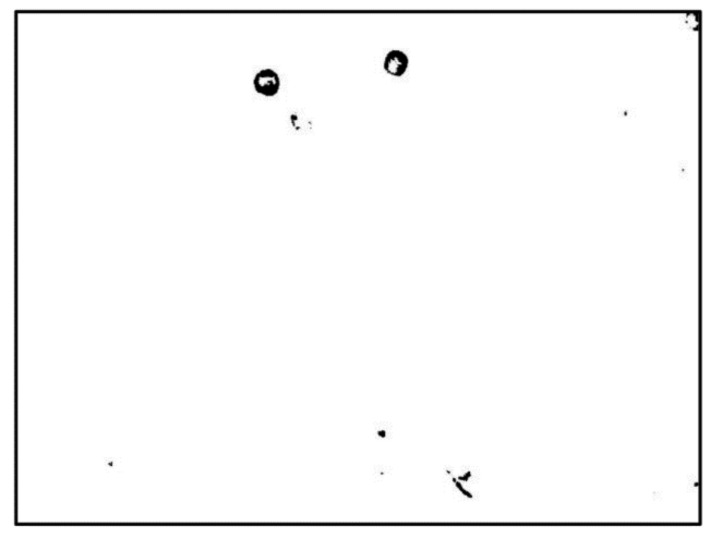
Image binarized using the OTSU method.

**Figure 8:**
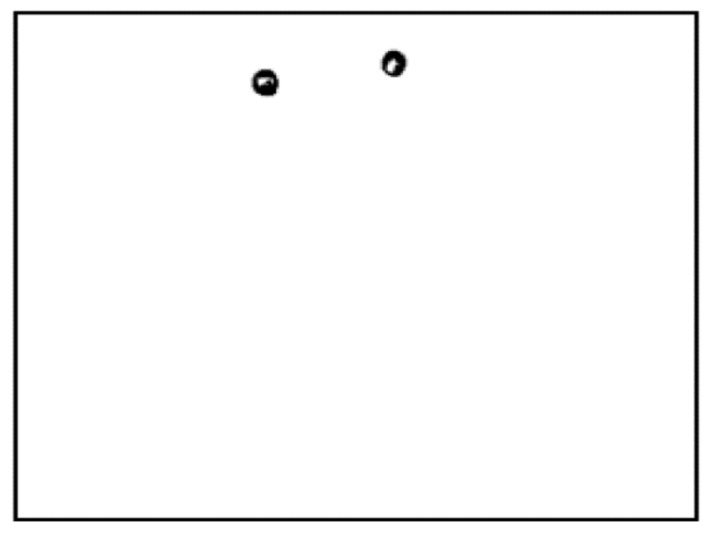
Complete cell outline after the image was denoised using the morphological method.

**Figure 9:**
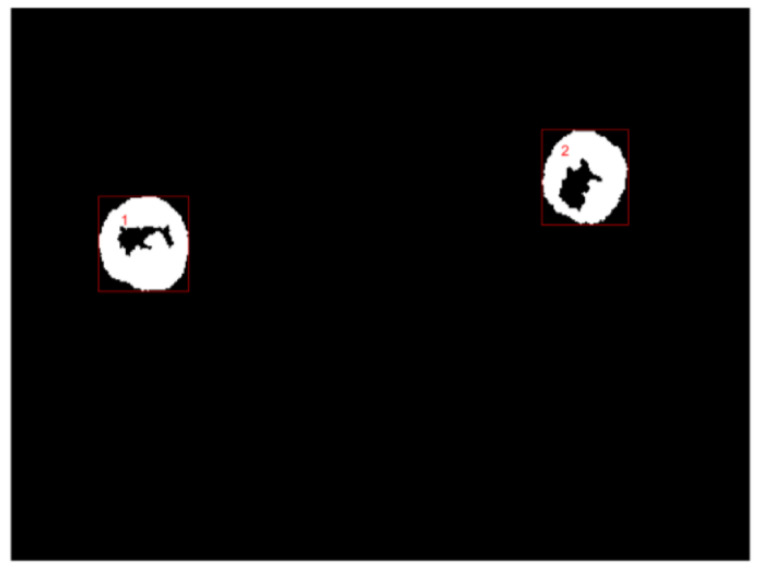
Cell identification and labelling.

**Figure 10:**
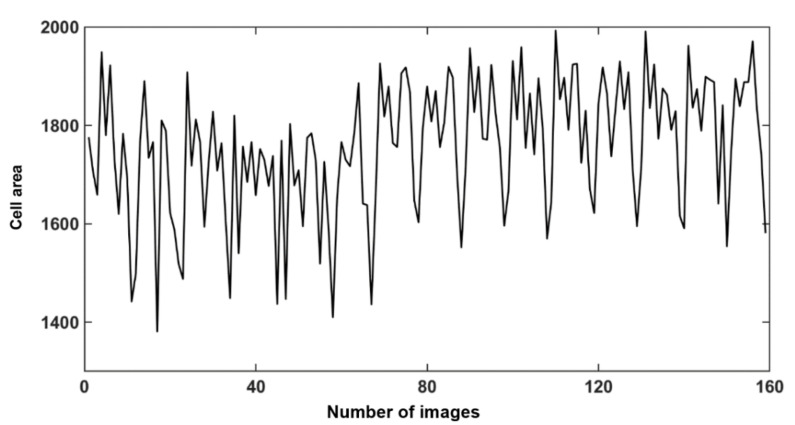
Area change curve based on the area of the cell of interest calculated in 159 images.

**Figure 11:**
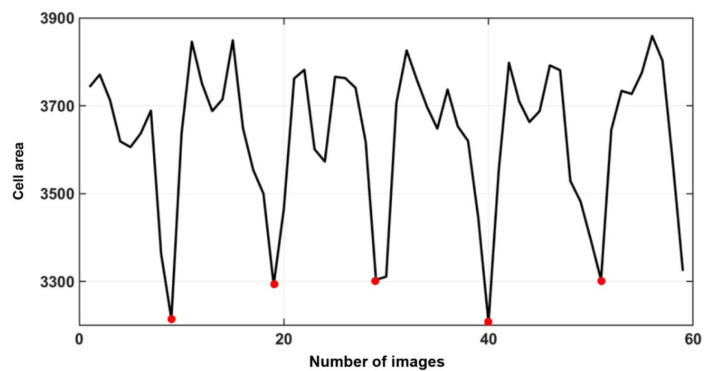
A smooth area change curve obtained based on data selected from the last 59 frames, which were then convolved and denoised.

**Figure 12:**
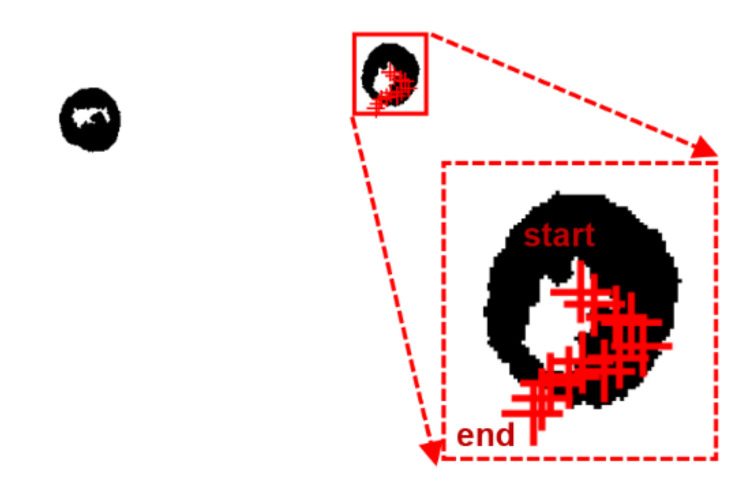
Motion trajectory of the cell of interest.

**Figure 13:**
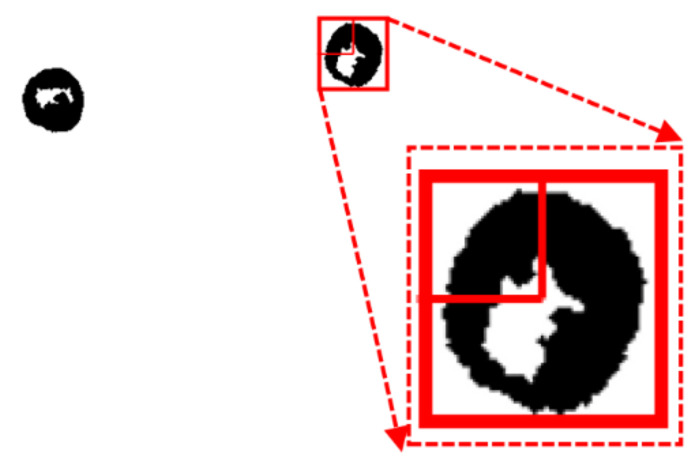
A method for calculating the cell area when the plane is rotated.

In our experiment, the bias voltage was set to 10 V_PP_ and Raji cells with a diameter of approximately 12 µm were observed at a 70 kHz AC frequency. A total of 159 cell images were acquired using a CCD camera, and the last 59 frames of five cycles of stabilization were used to calculate the average rotation speed. The original image and the image with dark corners removed are presented in Figure 5. Figure 6 shows the effect of a contrast-enhanced image. The image was binarized using the Otsu method, as shown in Figure 7. Figure 8 shows the way that the cell was identified and labeled, and Figure 9 shows a selected cell of interest. Figure 10 shows the area change curve obtained based on the area calculated for all of the frames. The data selected from frames 101 to 159 were convolved and denoised to obtain a clear area cycle change curve with an average self-rotation speed of 86 rpm over five cycles, as shown in Figure 11. The motion trajectory of the cell of interest is shown in Figure 12. The method for detecting the cell area when the cell was in-plane rotation is shown in Figure 13.

### 4.2. Self-Rotation Speed of Raji cells under Different AC Bias Parameters

**Figure 14:**
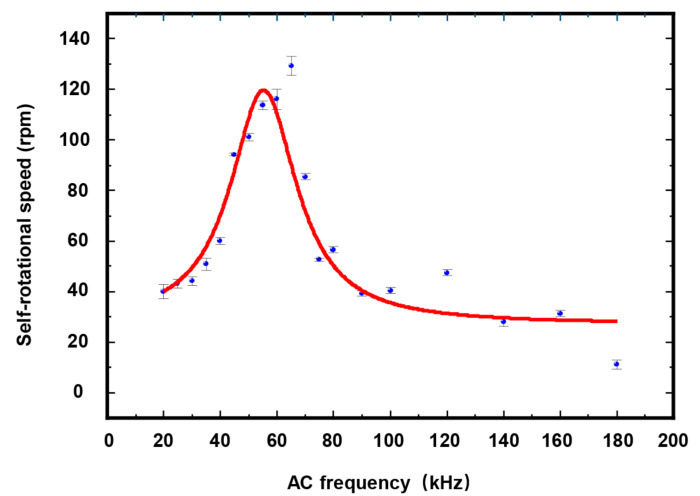
Self-rotational speeds of Raji cells with different AC frequencies at an external AC bias potential of 10 V_pp_. The measurement points in this figure were obtained for one Raji cell only. At each measurement point, the period of stable continuous cell rotation was selected for measurement, and the number of cell rotation periods was {3, 3, 5, 6, 4, 5, 5, 5, 5, 3, 5, 6, 5, 5, 3, 5, 2, 2, 2, 2, 2, 3, 2}. In the figure, each data point represents a mean ± maximum deviation.

**Figure 15:**
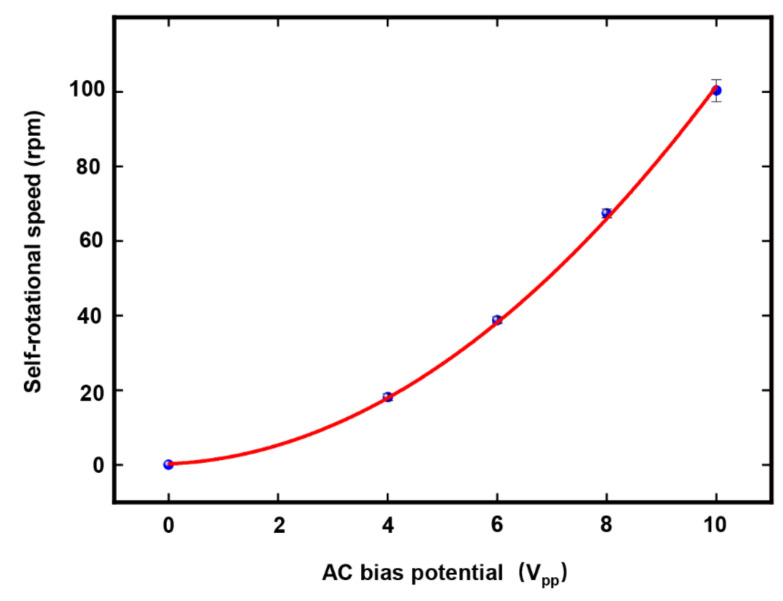
Self-rotational speeds of Raji cells with different AC bias potentials at an external AC frequency of 60 kHz. The measurement points in this figure were obtained for one Raji cell only. At each measurement point, the period of stable continuous rotation of the cell was selected for measurement, and the number of rotation periods for the applied AC bias potential of {4, 6, 8, 10} VPP was {1, 2, 3, 5}, respectively. In the figure, each data point represents a mean ± maximum deviation.

By varying the applied AC frequency and bias potential, it was possible to characterize the change in the self-rotation speed of Raji cells under different AC bias parameters. Figure 14 shows the self-rotation speeds of Raji cells as a function of different AC frequencies at a voltage maintained at 10 Vpp, using only one Raji cell for the measurements. The Raji cell was capable of self-rotation motion at a frequency scope ranging from 20 kHz to 180 kHz. The extracted self-rotation speed of the Raji cell proved an increase from 20 kHz to 65 kHz and then the self-rotation speed reached a maximum value of 129.4 rpm at 65 kHz. Furthermore, the characterization of the self-rotation speed of the Raji cells with respect to the AC bias potential was also investigated, as shown in Figure 15. In this figure, the AC frequency was maintained at 60 kHz. The results demonstrated a quadratic relationship between the self-rotation speed of the Raji cells and AC bias potential.

### 4.3. Discussion

The algorithm provides an effective method to calculate the self-rotation speed of cells in an ODEP field and to depict the motion trajectory of the cells. It can extract the rotation speed of cells quickly and accurately under different experimental conditions. All of the steps, except the selection of cells of interest, can be done automatically, eliminating interference with the experimental results through manual intervention. For cells with a homogeneous structure, the algorithm can calculate their rotation speed based on changes in their internal structure. In the case of in-plane rotation, the algorithm can also offer a highly adaptable and stable method for calculation. In the future, the algorithm can potentially provide some new ideas for the fast calculation of other rotational targets and rotational methods. The algorithm can also be combined with various microfluidic rotation platforms to expand its scope of application. For example, it can be used with a number of structure-based, acoustic-based, or optical-based microfluidic platforms for cell manipulation, which involve rotation and translation to extract the physiological properties of cells from their behavioral information [5,6,7,8,9,10,11,12,13,14,15,16,17,18,19,20,21,22,23,24,25,26,27,28,29,30,31,32,33,34,35,36,37,38,39,40,41,42,43,44,45,46,47,48,49,50,51,52,53,54,55,56]. Capturing cells of interest when they are rotating with high-speed translation will be a challenge that can, for example, be addressed by increasing the frame rate of cameras. In addition, multi-target rotation speed detection should be a priority area of research in future studies.

## 5. Conclusions

In this study, an area change algorithm was presented for the accurate extraction of self-rotation speeds and translational motions of cancer cells suspended in an ODEP chip, i.e., an AI-powered optical−electrical−microfluidic platform. The algorithm works well with microscopic images of cells under poor lighting conditions and with low contrast. The algorithm enables the automatic identification of cells and the extraction of their self-rotation speed. With a pre-processing stage that removes dark corners for increased contrast, the algorithm can clearly extract the cell outline, proving to be effective in processing cell micrographs under poor lighting conditions and with low contrast. A convolution method is used to process the area change curve, from which the cell rotation period can be determined clearly to help measure the self-rotation speed of the cells more accurately. The algorithm can process spherical and flat cells in the same image rotating at different angles by extracting the outline of the internal cell structure. In addition, it has a strong capability for processing cells rotating in-plane and out-of-plane. Furthermore, the algorithm can obtain the motion trajectory of cells of interest to allow for a more comprehensive analysis of the morphological and behavioral information of cells. Potential applications of this algorithm include separating different types of cancer cells in different populations, distinguishing normal cells from cancer cells, and characterizing the morphological and electrical properties of cells.
